# Reliability and validity of the Automatic Cognitive Assessment Delivery (ACAD)

**DOI:** 10.3389/fnagi.2014.00034

**Published:** 2014-03-06

**Authors:** Elisa Di Rosa, Caoimhe Hannigan, Sabina Brennan, Richard Reilly, Viliam Rapčan, Ian H. Robertson

**Affiliations:** ^1^Department of General Psychology, University of PadovaPadova, Italy; ^2^Trinity College Institute of Neuroscience, Trinity College DublinDublin, Ireland; ^3^Trinity Centre for Bioengineering, Trinity College DublinDublin, Ireland; ^4^School of Psychology, Trinity College DublinDublin, Ireland

**Keywords:** online assessment, monitoring, sustained attention, recognition memory, elderly, cognitive assessment battery

## Abstract

In this study we evaluated reliability and validity of the Automatic Cognitive Assessment Delivery (ACAD): a short computerized battery composed by memory and attention tests, delivered online, and designed primarily for the elderly. Reliability was examined with a test–retest design and validity was assessed by means of comparison with standard neuropsychological tests. Older (*N* = 32) and young adult participants (*N* = 21) were involved. We found that the ACAD is free from any practice effect. Test–retest reliability was confirmed via significant correlations and high percentage agreements between the scores of three repeated assessments. ACAD scores were lower for older than for young adult participants and correlated significantly with the standardized measures of memory and attention. Results demonstrate that the ACAD battery provides a reliable and valid measure of both immediate and delayed recognition memory and sustained attention, and may be useful for convenient and efficient cognitive assessment and monitoring in older adults.

## INTRODUCTION

Demographic aging is a global phenomenon that poses formidable socio-economic and health challenges, and results in a growing demand for efficient healthcare and support services to meet the needs of older adults. Perhaps one of the most significant challenges associated with an aging population is the increasing incidence of cognitive impairment with age. The development of accurate methods for monitoring cognitive functions in the elderly is of crucial importance in addressing this challenge. Such monitoring tools will allow for the detection of changes in cognitive functions, but also can be used to assess the effects of therapeutic or preventative interventions (Wild et al., 2008).

A review of emerging healthcare technologies by Hanson et al. (2013) noted that electronic monitoring systems provide opportunities to learn about the aging process and the progression of age-related diseases, and can contribute to new care models that promote the independence of older, at-risk adults in the home. For these reasons, a number of new methods for the assessment and monitoring of cognitive functions in the elderly have been developed (Dwolatzky et al., 2003; Fillit et al., 2008; Brouillette et al., 2013; Onoda et al., 2013), largely based on computer or mobile phone technology (Dwolatzky et al., 2003).

One limitation of tools currently available is that repeated assessment of the same individuals, particularly in the domain of episodic memory, is limited by the need to have multiple parallel versions of the repeated test. The use of the same material for repeat testing leads to practice effects, and performance on repeat administration can therefore reflect material-specific learning rather than core episodic memory function. Limited availability of parallel test forms can therefore restrict the usefulness these tools for continuous assessment and monitoring of cognitive functions over time.

In an attempt to address this issue, we devised a novel form of a word and shape immediate and delayed recognition memory task, where the to-be-remembered items were randomly selected from the same single master list on each testing occasion. It was hypothesized that proactive interference (Peterson and Gentile, 1965) from previous testing sessions would ensure that no practice effects occurred. It was also hypothesized that these simple, completely self-administered and highly usable repeatable recognition memory tests could be used for an indefinite number of assessment sessions.

In addition to these recognition memory tests, we included a go/no-go non-verbal shapes task, with a low probability no-go target shape, to which respondents have to withhold a response. This go/no-go non-verbal shapes task has some similarities to a validated test of sustained attention – the Sustained Attention to Response Test (SART) (Robertson et al., 1997). The SART was found to be free of practice effects and to measure important aspects of this key cognitive capacity. The inclusion of this last test in the current assessment also served to fill the interval between immediate and delayed recall of the word and shape items.

The two recognition memory tests and the sustained attention test were incorporated into a short computerized battery, the Automatic Cognitive Assessment Delivery (ACAD), an instrument developed for remote delivery using a dedicated website (https://acad.tchpc.tcd.ie/). This web-based instrument allows participants to register for an individual account, automatically generated using the same website interface, and to then easily undertake the cognitive assessment at home, which takes no longer than 15–20 min. The nature of the tests included, explained in detail in the next section, together with the relative brevity of the battery and the availability of the instrument online, make the ACAD a potentially useful method for repeated cognitive assessment and monitoring.

In the current study we evaluate the feasibility of the ACAD, examining reliability with a test–retest design and assessing validity by means of comparison with standard neuropsychological tests. Finally, this study involved both older and young adult participants in order to investigate the sensitivity of the ACAD to age related differences in memory and attention.

## METHOD

### PARTICIPANTS

A total of 59 volunteers were recruited to take part in this study. Participants in the older adult group were recruited from a panel of research volunteers at Trinity College Institute of Neuroscience. Participants in the young adult group were recruited through public advertisements and contact with students in the School of Psychology at Trinity College Dublin, who received undergraduate research credits in return for participation.

Six participants dropped out after the first assessment, and 13 dropped out after the second assessment. Therefore data for this study comprised that from a total of 53 participants who completed two assessments, and 40 participants with all the three assessments completed. Thirty-two (14 male) older (age range 65–77; mean = 68.87; SD = 3.05) and 21 (5 male) young (age range 18–35; mean = 25.15, SD = 2.14) adults completed the online elements of the study. Of the 32 older adults, 20 (8 male) participants (age range 65–77; mean = 68.65; SD = 3.15) also completed a neuropsychological assessment session (see **Table 1**). Inclusion criteria for this study were participants with normal or corrected to normal vision, who were native English speakers and living independently. Exclusion criteria were the presence of neurological disease (any medical conditions associated with a head injury, epilepsy, stroke), reported history of psychiatric disorder or neurological disease, and use of psychiatric and neurological medications. Written informed consent was obtained from all participants. The ethics committee of the School of Psychology, Trinity College Dublin, approved the study.

**Table 1:** Characteristics of the samples recruited for this study.

Sample	Age (years)	Years of education	NART estimated IQ *N* = 20	MoCA total score *N* = 20	CES-D total score *N* = 20
Older *N* = 32	68.87 ± 3.05	17.28 ± 1.3	119.36 ± 5.4	27.15 ± 1.9	6.3 ± 3.9
Young *N* = 20	25.6 ± 2.8	18.65 ± 1.3	–	–	–

*Tests mean scores refer to the sample that attended the neuropsychological assessment for the validity analysis. NART, National Adult Reading Test, MoCA, Montreal Cognitive Assessment; CES-D, Centre for Epidemiologic Studies Depression Scale.*

### PROCEDURE

Participants were contacted by phone or email to confirm their interest in taking part in the study. Each participant then received an email containing a link to the ACAD web page, and details of their individual account for completion of the online assessment. When participants first visited the website and logged in to their account, they were asked to complete the online consent form, before proceeding to the assessment. Participants were required to complete the ACAD battery on three occasions – once a week for a period of 3 weeks. Each assessment lasted for approximately 15–20 min. Upon completion of the online assessment, participants were advised of the date of their next assessment, which was exactly 7 days later. An email reminder was also sent to every participant, one day before the date of each online assessment. Participants in the older adult group were also invited to attend a neuropsychological assessment session at Trinity College Institute of Neuroscience, after the completion of the online assessment. This session consisted of a battery of standardized neuropsychological measures, and took no longer than one hour to complete.

### MEASURES

#### Automatic cognitive assessment delivery

The ACAD online assessment was developed in Trinity College Dublin by Viliam Rapčan, Ian H. Robertson, and Richard Reilly. The battery comprises two memory tests, verbal and non-verbal, and one attention test. Verbal and non-verbal memory test contain an immediate and delayed recognition phase. The attention test, which consists of an adapted version of the SART (Robertson et al., 1997), was positioned between the immediate and delayed recognition phase of the memory tests.

**FIGURE 1:**
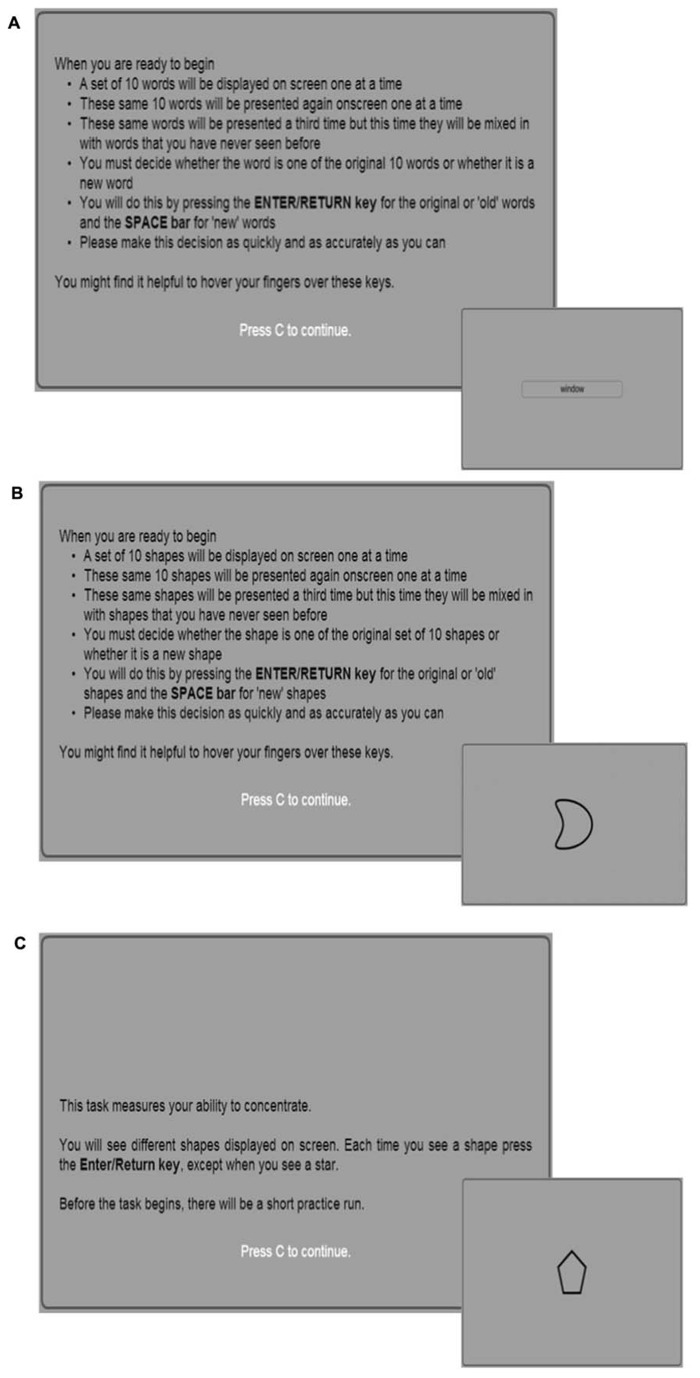
**Graphical interfaces of the three tests included in the ACAD battery. (A)** Instructions and example of stimulus of the Word Recognition test. In the presentation phase, every word was displayed at the center of the screen for 2000 ms, followed by a 1000 ms pause during which the screen was blank. In the recognition phase, the randomized list of 20 words was presented one by one until the participant response. **(B)** Instructions and example of stimulus of the Shape Recognition test. In the presentation phase, every shape was displayed at the center of the screen for 2000 ms, followed by a 1000 ms pause during which the screen was blank. In the recognition phase, the randomized list of 20 shapes was presented one by one until the participant response. **(C)** Instructions and example of non-target stimulus presented in the Shapes Sustained Attention to Response Task (SSART). Both types of stimuli remained on screen for 500 ms, with 1500 ms inter stimulus interval. The non-targets were presented 85 times; the target, a star, was displayed 15 times and it was presented one by one in random order with at least two non-target shapes being displayed between.

***Word recognition task.*** Two phases compose the word recognition task: immediate and delayed recognition. A list of 20 nouns with mean length of 4.95 letters (SD = 1.36, range: 3–8) and mean normative frequency of 82.45 (SD = 108.44) occurrences per million in The Sydney Morning Herald word database (Davis, 2005), was selected for inclusion at both the immediate and delayed recognition phase. The word recognition immediate (WRI) phase consisted of a presentation and recognition section. During the presentation section of the task, a list of 10 words, randomly selected from the pool of 20 nouns described above, were displayed one at a time on screen. Participants were presented with this list of 10 words twice in a randomized order. Each word was displayed at the center of the screen for 2000 ms, followed by a 1000 ms pause during which the screen was blank. During the recognition section of the task, all 20 words were randomized again and presented one by one at the center of the screen. Participants were required to indicate whether the displayed word was from the original list (displayed during presentation) or a new word (from the ten words that were not displayed during presentation), by pressing one of two keys which represented an “old” or “new” word (see **Figure 1A**). The word recognition delayed (WRD) phase included only the recognition section of the task, in which the list of 20 words was randomized again. In this second phase, participants were required to answer whether the displayed word was from the original list (displayed during WRI presentation) or a new word, again by pressing one of two keys on their computer keyboard.

***Shape recognition task.*** The shape recognition task, with the immediate (SRI) and delayed (SRD) phases, followed the same procedures as the two phases of the word recognition task (WRI and WRD). However, instead of words, this task employed 20 abstract shapes, generated in a manner that would not evoke association with words or common objects of the real world (see **Figure 1B**).

***The shapes sustained attention to response task.*** To measure participant’s sustained attention, the Shapes Sustained Attention to Response Task (SSART) was employed. This test is considered as a version of the SART (Robertson et al., 1997), adapted for use on the study’s website. The SSART required participants to press a key to frequently displayed non-targets and to withhold motor responses to occasional targets (see **Figure 1C**). The non-targets were represented by eight different shapes and were presented 85 times; the target was represented by the shape of a star and was displayed 15 times, presented one by one in random order with at least two non-target shapes being displayed between. Both types of stimuli remained on screen for 500 ms, with 1500 ms inter stimulus interval.

#### Standard neuropsychological tests

In order to assess the concurrent validity of the ACAD, participants in the older adult group were invited to attend a testing session at Trinity College Dublin, and complete a battery of standardized neuropsychological tests. 20 participants of the older group were evaluated with the following standardized measures: the Montreal Cognitive Assessment (MoCA; Nasreddine et al., 2005), used as a brief screen for cognitive impairment; the National Adult Reading Test (NART; Nelson, 1982), which provides an estimate of premorbid intellectual ability; Memory abilities were assessed by logical memory I and II subtests of the Wechsler Memory Scale (WMS-IV – UK; Wechsler, 2008) and by the Rey/Osterrieth Complex Figure task (Rey, 1941; Osterrieth, 1944).

Finally, to measure attention and executive functions, category fluency (animal) task, and the original version of the SART-fixed (Robertson et al., 1997) were also administered.

Mood and psychological stress were evaluated with the Centre for Epidemiologic Studies Depression Scale (CES-D; Radloff, 1977) and the anxiety subscale of the Hospital Anxiety and Depression Scale (Zigmond and Snaith, 1983).

### DEPENDENT VARIABLES

The ACAD automatically calculated and provided the raw scores for each task. For the SSART, mean reaction times (RT), standard deviation of RT (SD), coefficient of variation of RT (CoV), and number of commission errors were provided for every participant. We considered in the analysis the first three values and, to control the speed-accuracy trade-offs, we divided the number of commission errors by the mean RT (Seli et al., 2013). For the memory tasks, the raw score automatically calculated consisted in the number of words and shapes correctly recalled (from 0 to 20) in the immediate and delayed recognition sections of every memory task. These scores were then scaled considering the SD of our sample: for the WRI and SRI, given a mean SD of 2, we considered the performance as following: good if the raw score was between 20 and 16; low if the score was between 15 and 11; invalid if the score was equal or less to 10. For the WRD and SRD, given a mean SD of 2.5, we considered the performance as good if the raw score was between 20 and 15, low if the score was between 14 and 11, and invalid if the score is equal or less to 10.

## RESULTS

Data from a total of 53 participants, who completed the first two weekly assessments, were analyzed to evaluate the presence of a practice effect between the first and the second week. To evaluate the presence of a practice effect between the second and the third week data of 40 participants were compared. The reliability was evaluated between the first and the second (*N* = 53) and between the second and the third (*N* = 40) assessments. The concurrent validity was evaluated comparing the first week ACAD scores and the performance at the standard neuropsychological assessment (*N* = 20). Given that the normality tests (Shapiro–Wilk and Kolmogorov–Smirnov tests) indicated that not all the variables were normally distributed, non-parametric analyses were performed on the data. Two outlier participants of the older group were eliminated from the analysis of the commission errors (number of errors >80).

### PRACTICE EFFECT

**FIGURE 2:**
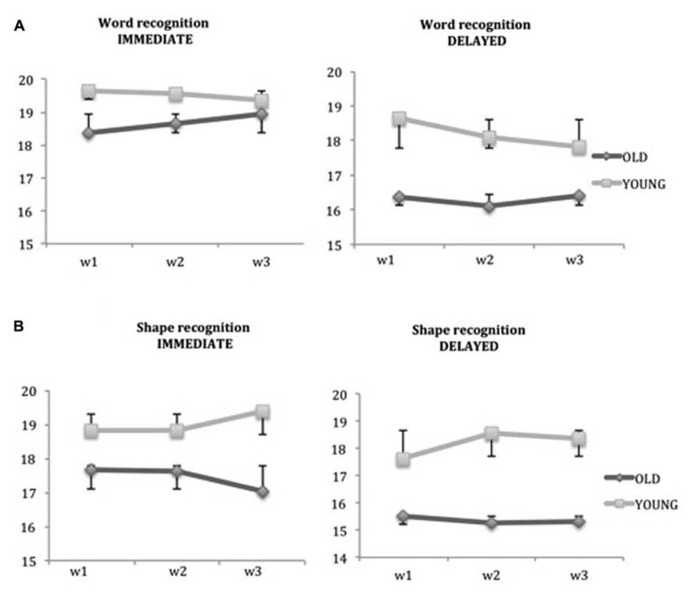
**Absence of learning effects in the verbal memory tests. (A)** Immediate and delayed Word Recognition test. **(B)** Immediate and delayed Shape Recognition test. Mean scores and standard deviations of each assessment. Non-significant learning effects were found between the three assessments, in both young and old groups. In the first two weeks w1: week 1; w2: week 2; w3: week 3.

To test the presence of a practice effect, a Wilcoxon signed-rank test was performed using the raw scores of every assessment, separately for each group. In the analysis of the recognition memory tests (**Figure 2**) no significant difference was found between the scores obtained in each assessment. Both older and young adults did not show any practice effect in the word immediate and delayed recognition (**Figure 2A**) and in the shape immediate and delayed recognition (**Figure 2B**).

**FIGURE 3:**
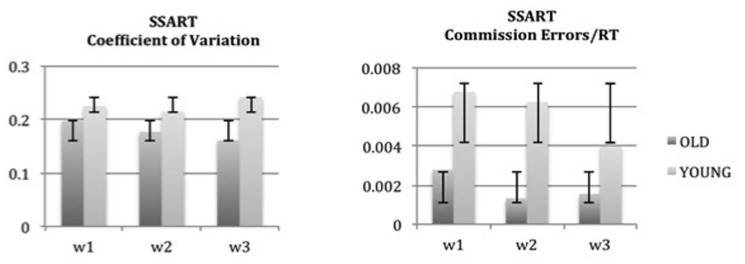
**Absence of learning effects in the Shapes Sustained Attention to Response Task (SSART).** Mean scores and standard deviations of each assessment are reported for coefficient of variation and commission errors of the SSART. Non-significant learning effects were found between the three assessments, in both young and old groups. w1: week 1; w2: week 2; w3: week 3.

Similar results were obtained in the analysis of all the measures considered for the SSART. As **Figure 3** shows, the absence of a practice effect in both groups is evident also in the key measures of the SSART, CoV, and commission errors. The non-significant differences found in every analysis (*p* > 0.05), indicate a clear absence of a practice effect for the ACAD measures, in both older and young adults.

### RELIABILITY

The reliability of the ACAD was evaluated using a Spearman’s correlation analysis, between raw scores of the first and the second assessment and between the raw scores of the second and the third assessment (**Table 2A**). Given the scoring procedure used for the word and the shape recognition tests the reliability of the memory test was also evaluated calculating the percentage agreement between the scaled scores of each assessment (**Table 2B**). The percentage agreement procedure was used in order to avoid the influence of the scaling procedure on the correlation analysis (Poulton, 1968). Significant test–retest reliability was evident in nearly all measures. The high scores obtained by our sample in the WRI indicate the presence of a ceiling effect in this memory test, which could explain a significant but low correlation between week 1 and week 2 performances, and a non-significant correlation between week 2 and week 3 performances. The very high percentage agreements calculated between the scaled-scores of the same test (**Table 2B**) confirm that the correlation coefficient was biased by the distribution of scores on the measure.

**Table 2A:** Test–retest reliability: spearman correlations between first and second and between second and third ACAD administrations.

Test	Week 1 mean ± SD (*N* = 53)	Week 2 mean ± SD (*N* = 53)	Week 3 mean ± SD (*N* = 40)	Correlations week 1–week 2 (*N* = 53)	Correlations week 2–week 3 (*N* = 40)
WRI	18.19 ± 4.34	18.98 ± 1.47	19.05 ± 1.03	0.353 ± 0.009*	0.089 ± 0.586
WRD	17.58 ± 2.58	17.13 ± 2.46	16.88 ± 2.37	0.334 ± 0.014*	0.352 ± 0.026*
SRI	18.19 ± 1.69	18.21 ± 1.75	17.75 ± 2.14	0.477 ± 0.000*	0.430 ± 0.006*
SRD	16.58 ± 2.43	16.40 ± 2.38	16.17 ± 2.92	0.452 ± 0.000*	0.607 ± 0.000*
SSART mean RT	432.65 ± 116.07	443.41 ± 121.3	439.44 ± 81.42	0.797 ± 0.000*	0.821 ± 0.000*
SSART CoV	0.20 ± 0.08	4.8pt0.19 ± 0.06	0.19 ± 0.08	0.413 ± 0.002*	0.805 ± 0.000*
SSART St.Dev	89.90 ± 46.87	83.10 ± 27.79	80.01 ± 29.56	0.433 ± 0.001*	0.592 ± 0.000*
SSART commission errors/RT	0.0045 ± 0.004	0.0036 ± 0.005	0.0025 ± 0.003	0.563 ± 0.000*	0.694 ± 0.000*

WRI, Word recognition immediate; WRD, Word recognition delayed; SRI, Shape recognition immediate; SRD, Shape recognition delayed. SSART CoV, Shapes Sustained Attention to Response Task Coefficient of Variation. *Statistically significant correlations refer to a *p* value <0.05, based on Spearman’s correlation coefficient.

**Table 2B:** Percentage agreements of memory tests scaled scores, between first and second and between second and third ACAD administrations.

Test	Week 1–Week 2 (*N*=53)	Week 2–Week 3 (*N*=40)
WRI	87%	92.5%
WRD	75%	75%
SRI	88%	73%
SRD	79%	77.5%

### VALIDITY

For the concurrent validity analysis, we performed Spearman’s rank correlations in order to examine the relationship between ACAD scores from the first assessment and scores on standardized cognitive measures, administered during the assessment session at TCD.

As **Table 1** shows, the sample considered did not exhibit evidence of dementia or depression and also had an above average estimated IQ. The analysis was performed using raw scores for all standardized tests, apart from the Wechsler Logical Memory tests, for which we used percentage scores when investigating correlation with the ACAD memory tests^1^.

The results show a good correlation between each ACAD scores and the standardized measures of memory and attention. In detail, the WRI has a good correlation with the immediate recall of the Wechsler Logical Memory (*r* = 0.479; *p* < 0.05), with the MoCA total score (*r* = 0.478; *p* < 0.05) and with the visuospatial-executive subscale of the MoCA (*r* = 0.465; *p* < 0.05). The WRD has a good correlation with the Rey figure recall (*r* = 0.448; *p* < 0.05), with the visuospatial-executive and the delayed recall subscales of the MoCA (respectively, *r* = 0.466 and *r* = 0.452; *p* < 0.05), and with the MoCA total score (*r* = 0.529; *p* < 0.05). The SRI and SRD have a good correlation with the Rey figure recall (respectively, *r* = 0.610 and *r* = 0.546; *p* < 0.05) and with the NART estimated IQ (*r* = 0.462 and *r* = 0.507; *p* < 0.05). The SRD also have a good correlation with the immediate recall (*r* = 0.516; *p* < 0.05) and delayed recognition (*r* = 0.619; *p* < 0.05) of the Wechsler Logical Memory. Only the SRI correlates with the CES-D (*r* = -0.566; *p* < 0.01).

For the attention task, we found good correlations between the SSART and the SART-fixed. In detail, the mean RT of the SSART is significantly correlated with the SD of the SART-fixed (*r* = 0.540; *p* < 0.05); the CoV of the SSART is correlated with the CoV of the SART-fixed (*r* = 0.537; *p* < 0.05). The SD of the SSART is correlated with the CoV (*r* = 0.445; *p* < 0.05) and with the SD of the SART-fixed (*r* = 0.612; *p* < 0.01). The CoV of the SART-fixed is also correlated with the the commission error (*r* = 0.571; *p* < 0.01) of the SSART. The SD of the SSART was also found to be significantly correlated with the Rey figure recall scores (*r* = 0.493; *p* < 0.05). Finally, considering the entire sample data at week 1, we found that none of the ACAD scores are correlated with education.

### GROUP DIFFERENCES

With the aim to investigate the sensitivity of the ACAD to age related differences in memory and attention, we firstly performed a correlational analysis between ACAD scores and age of participants. Results show that age is significantly correlated with the most of the ACAD scores: WRD (*r* = -0.428; *p* < 0.01), SRI (*r* = -0.296; *p* < 0.05) SRD (*r* = -0.357; *p* < 0.01), Mean RT (*r* = 0.541; *p* < 0.01) and commission errors of the SSART (*r* = -0.310; *p* < 0.05). Given these results, a Mann–Whitney *U* test was performed on the first week assessment scores to assess the group differences. The results show a significant difference between the two groups, young and older adults, in the WRI (*U* = 438; *p* < 0.05), WRD (*U* = 540; *p* < 0.01), SRI (*U* = 476; *p* < 0.01) and SRD (*U* = 518.5; *p* < 0.01). Commission errors and mean RT of the SSART were significantly different between young and older (respectively, *U* = 465 and *U* = 114; *p* < 0.01), and no group differences were found for the SSART SD and the SSART CoV. The same significant differences were found performing the same analysis with the scores of week 2.

## DISCUSSION

The principal goal of this study was to assess whether the ACAD, a short cognitive assessment battery delivered online in the participant’s home, was a valid measure for the assessment of cognitive functions. Furthermore we wanted to test the reliability of the ACAD, a battery free of parallel versions, to test the usability of this instrument for repeated assessment practice.

The results of this study show that the ACAD is a reliable and valid instrument, free from learning effects. The absence of learning between two assessments, separated by an interval of only 7 days, suggests the utilization of the ACAD for the monitoring of cognitive functions, predisposing the evaluations with intervals of at least 1 week. These results support also the possibility of using the first online assessment as a practice session, before the real assessment a week later. Adopting this approach increases reliabilities of most tests.

The results of reliability analyses show a good reliability between the three weekly assessments considered in this study, for almost every ACAD scores. The low correlation values between some scores in this study may be explained in part by the high-performing characteristics of the sample. A possible ceiling effect may have biased the correlation index used for the evaluation of reliability. In fact, reliability was increased by assessing the stability of scores within bands, defined by the mean standard deviation calculated in these three assessments for each memory test. Using these values, we defined the performance as good or low, if the score was within two standard deviations and invalid if the score was below the chance level. The evaluation in bands of the ACAD scores may be a useful method for remote monitoring of cognitive functions over time. In this way, a significant change in performance from session to session (i.e., moving from one band to another) could be easily and automatically detected.

The results of this study also suggest that the ACAD battery provides valid measures of attention and recognition memory. The significant correlations observed between ACAD scores and standard neuropsychological tests suggest that ACAD may be a useful and convenient battery for monitoring recognition memory and attention over time in the elderly. This study also provides support for the feasibility and convenience of using an internet-based battery, such as the ACAD, to assess and monitor cognitive function over time. Our participants had varying levels of familiarity with computers, particularly in the older group, but did not report any major difficulties using this online assessment, which has clear instructions and simply involves pressing one or two buttons on the computer keyboard. This demonstrates the suitability of the ACAD for assessment of an elderly population without high confidence and limited experience computer technology.

Home-based online assessments may be beneficial for both participants and researchers. Participants can experience a low stressing and more comfortable setting for the assessment. Researches have a time-efficient and convenient assessment instrument: they can have a valid and reliable evaluation of memory and attention, in spite of the variable conditions of a home assessment. Furthermore, online assessment using ACAD allows the researcher to collect valid measures of sustained attention and immediate delayed recognition memory, from a large number of participants in a short time period. Finally, the detailed results of each test are available to the researcher immediately after each administration, avoiding time consuming scoring and analysing procedures.

In summary, this study has demonstrated that the ACAD battery provides a reliable, valid and repeatable measure of both immediate and delayed recognition memory, and sustained attention. ACAD may be a useful instrument for the convenient and efficient assessment and monitoring of cognitive functions in older adults. Further studies with clinical samples will allow for evaluation of the suitability of the ACAD in assessment and rehabilitation procedures after illness.

## Conflict of Interest Statement

The authors declare that the research was conducted in the absence of any commercial or financial relationships that could be construed as a potential conflict of interest.

## Footnotes

1 The different forms of the Wechsler Logical Memory test, used depending on the age of the participant, have a different and not equivalent range of raw scores.
